# Endogenous alpha-synuclein monomers, oligomers and resulting pathology: let’s talk about the lipids in the room

**DOI:** 10.1038/s41531-019-0095-3

**Published:** 2019-11-12

**Authors:** Bryan A. Killinger, Ronald Melki, Patrik Brundin, Jeffrey H. Kordower

**Affiliations:** 1 Department of Neurological Sciences, Rush University Medical Center, Chicago, IL 60612 USA; 2 CEA and Laboratory of Neurodegenerative Diseases, Institut Francois Jacob (MIRCen), CNRS, 92265 Fontenay-Aux-Roses cedex, France; 3 Center for Neurodegenerative Science, Van Andel Research Institute, Grand Rapids, MI 49503 USA

**Keywords:** Neuroscience, Molecular biology

## Abstract

Alpha-synuclein is an intrinsically disordered, highly dynamic protein that pathogenically aggregates into inclusion structures called Lewy bodies, in several neurogenerative diseases termed synucleinopathies. Despite its importance for understanding disease, the oligomerization status of alpha-synuclein in healthy cells remains unclear. Alpha-synuclein may exist predominantly as either a monomer or a variety of oligomers of different molecular weights. There is solid evidence to support both theories. Detection of apparent endogenous oligomers are intimately dependent on vesicle and lipid interactions. Here we consider the possibility that apparent endogenous alpha-synuclein oligomers are in fact conformations of membrane-bound alpha-synuclein and not a bona fide stable soluble species. This perspective posits that the formation of any alpha-synuclein oligomers within the cell is likely toxic and interconversion between monomer and oligomer is tightly controlled. This differs from the hypothesis that there is a continuum of endogenous non-toxic oligomers and they convert, through unclear mechanisms, to toxic oligomers. The distinction is important, because it clarifies the biological origin of synucleinopathy. We suggest that a monomer-only, lipid-centric view of endogenous alpha-synuclein aggregation can explain how alpha-synuclein pathology is triggered, and that the interactions between alpha-synuclein and lipids can represent a target for therapeutic intervention. This discussion is well-timed due to recent studies that show lipids are a significant component of Lewy pathology.

## Introduction

Alpha-synuclein (αSyn) is an intrinsically disordered, highly flexible protein, which plays an important role in the pathogenesis of several neurodegenerative diseases cumulatively referred to as synucleinopathies. In different synucleinopathies, neurons and/or glia bare the hallmark intracellular deposits of filamentous αsyn^1^ but the origin of this pathology remains unclear. αSyn oligomers with β-sheet structure (αsynO-β) are toxic to cells, possibly through physical disruption of cellular membranes.^2,3^ αSynO-β/preformed fibrils (PFFs) generated in vitro or isolated from the brains of patients developing synucleinopathies can “seed” aggregates, especially in transgenic rodent models overexpressing αsyn,^4^ and initiate a toxic cascade reminiscent of that seen in disease.^5–7^ However, not all oligomers are believed to be bad actors, as evidence suggests that various oligomers may not only exist in the cell but also have normal cellular functions.^8–10^ Indeed, some conformers of αsynO-β do not seed pathology and are non-toxic.^11–13^ Functional endogenous oligomers have been controversial, as their existence has been both confirmed and refuted by carefully executed studies.^8,14,15^

Are there benign αsyn oligomers that have normal cellular functions, and if so, how do they transition to toxic αsynO-β? Here, in this short review, we discuss how lipid–αsyn interactions might help explain the observation of apparent endogenous non-toxic oligomers and highlight alternative models that are monomer-centric. Furthermore, we will extend this viewpoint to consider its implications for synucleinopathy pathogenesis. There has been substantial work done in this area and several exhaustive reviews on lipid–αsyn interactions^16–21^ and oligomerization^22,23^ are available; hence, for the sake of clarity, we will not comprehensively discuss the literature.

## Biology of lipids in neurodegenerative disease

The brain is ~60% lipids by weight.^24^ Lipids have diverse cellular functions in biology including cell signaling, energy storage, and structural partitioning.^25^ Phospholipids contain amphipathic characteristics with a charged hydrophilic phosphate group and a carbon chain of varying lengths.^25^ Phospholipids spontaneously form bilayer structures in aqueous solutions that are the basis of cellular membranes. Lipids have not been as extensively studied as proteins in vivo, possibly because of their hydrophobicity, chemical complexity, and the fact that they are not gene products.^25^ However, lipids are crucial for cellular function and are implicated in several neurodegenerative diseases including synulceinopathies.^26^ Recent technological advances with lipodomic analysis have furthered the study of lipids.^27–30^ Current lipodomic analyses, however, are focused on whole-cell lysates and therefore insensitive to cellular spatial and temporal dimensions, which are crucial for understanding lipid function.^25^

## αSyn–vesicle membrane interactions

Shortly following the discovery of αsyn in Lewy pathology,^31^ the lipid-binding properties of asyn were documented and the significance of disease-causing point mutations in lipid-binding domains was recognized.^32^ Indeed, one of the earliest histochemical descriptions of Lewy bodies noted that they stain positively for phospholipids, particularly sphingomyelin.^33^ Since then, interactions between αsyn and vesicle lipids have been implicated in the initial generation of synucleinopathy.^16–19,34–36^ The hypothesis that lipids control pathological αsyn aggregation primarily stems from observations that lipids/vesicles influence aggregation kinetics in vitro,^34,37–40^ and that disease-related missense mutations of *SNCA* alter residues within the N-terminal lipid-binding domain of αsyn.^41–46^ αSyn may redistribute to lipid compartments early in disease pathogenesis.^36^ Several disease-causing αsyn mutants abnormally associate with intracellular vesicles and lipid droplets,^47,48^ and dyshomeostasis of intracellular lipids are likely an early molecular event preceding pathology formation.^49^ The key to pathogenesis lies within the lipid-binding domain of αsyn.

αSyn binding to vesicular membranes is important, because it influences oligomerization and pathological aggregation. Evidence suggests that non-pathological αsyn is involved with vesicular dynamics in cells^9,18,50–55^ and regulation of the presynaptic vesicle pool.^56,57^ αSyn–lipid interactions may have a vesicle tethering function. It has been proposed that the broken α-helical N-terminus can function to tether intracellular vesicles via a “double-anchor” mechanism.^58–61^ The biological significance of αsyn-mediated clustering is unknown; however, it could serve to promote the exchange of lipids between adjacent vesicles^62^ and possibly promote vesicle fusion.^56–58,62–64^ αSyn preferentially binds to membranes with lipid-packing defects^65–68^ and high curvature.^69–72^ In the neurons, αsyn is densely clustered around intracellular vesicles and vesicular tubule structures, most prominently at the nerve terminal.^73^ When incubated with small (~10–30 nm) unilamaller vesicles, the N-terminal of αsyn adopts an extended α-helical conformation as it coats the vesicular surface and a broken α-helical conformation when interacting with micelles.^59,74–80^ The interaction between the N-terminus of αsyn with lipid membranes is driven by electrostatic interactions between positively charged residues and lipid phosphate head group.^81^ When membrane bound, the N-terminus residues (1–26) of αsyn rigidly bind to the membrane and the internal segment (residues 26–97) acts to sense lipid properties and regulates binding affinity.^82^ Interestingly, the hydrophobic stretch of residues 71–82 are required for pathological aggregation of αsyn^83^ and, therefore, lipid-sensing properties of αsyn and pathological aggregation occur through the same functional domain. It is not clear whether mutation of the N-terminus results in a toxic gain of function or loss of function.

Many cellular functions have been attributed to αsyn and membrane interactions, including soluble N‐ethylmaleimide sensitive factor attachment protein receptor (SNARE) complex assembly and exocytosis; however, the exact cellular function of αsyn remains unclear.^9,50,84^ αSyn interacts with SNARE proteins at the vesicle surface.^85–88^ αSyn binding to membranes promotes SNARE complex formation and may function as a SNARE chaperone protein.^9,88,89^ Vesicular membrane-binding promotes the oligomerization of αsyn.^9^ In vitro phospholipids can also increase the rate of pathological aggregation (i.e., β-sheet confirmations) by decreasing lag time of primary nucleation.^37,90^ The effect of lipids on αsyn aggregation is dependent on lipid to protein ratio, with a low ratio promoting aggregation and higher ratio being inhibitory.^74,91^ This bimodal phenomenon probably results from a lack of monomer available for oligomer elongation when the lipid ratio is too high. Interestingly, increasing αsyn expression, presumably shifting the intracellular lipid to protein ratio, promotes aggregation of αsyn in cells. Notably, it has been hypothesized that reducing monomeric αsyn is an important therapeutic target.^92–94^

A confusing aspect to the literature is that binding of αsyn to membranes has been reported to both inhibit^95–97^ and to promote αsyn aggregation.^39,47^ This may be due to differences in assay conditions between studies, such as membrane lipid composition and αsyn concentration. Indeed, recent studies using lipodomics implicated specific fatty acid oleic acid in the pathogenesis of Parkinson’s disease.^49^ In the model proposed by Fanning and colleagues^49^, soluble αsyn binds to oleic acid, effectively sequestering the monomer to lipid membranes and ultimately culminating in pathological aggregate formation. As they also observed an increase in oleic acid in response to αsyn overexpression, there may be a toxic lipid dyshomeostatis that preceeds aggregate formation. Their results suggest a complex origin of synucleinopathy where both lipid metabolism and αsyn are central players.

αSyn may have a more generalized cellular function as an effector of lipid dynamics, and not as a factor of a specific subprocess or pathway. To highlight this concept, consider the curious relationship between αsyn and erythropoiesis (i.e., red blood cell differentiation) for which others have hypothesized αsyn that may have an underlying redundant mechanism in the two cells of different linage.^98^ αSyn is highly expressed in erythrocytes under the control of transcription factor GATA1.^99,100^ During the terminal step of erythropoiesis, α-syn expression dramatically increases and remains elevated in the mature erythrocyte.^98^ αSyn is then found associated with phospholipids and vesicle membranes in the mature erythrocyte.^101^ Thus, which of the proposed cellular functions does αsyn perform during erythropoiesis? One likely explanation is that αsyn plays a role in the dramatic intracellular lipid organization, analogous to asymmetric cytokinesis, which occurs prior to the phenomenon of enucleation. Enucleation is the process by which organelles are condensed and extruded from the cell to form a mature erythrocyte. Indeed, just prior to enucleation αsyn can be found associated with lipids of the cell, particularly the nucleus and ER, which are key players in enucleation. αSyn accumulates at the site of nuclear extrusion,^102^ suggesting it is directly involved with enucleation lipid dynamics. Concurrently, SNARE machinery is decreased in the erythrocyte, suggesting that the potential involvement of αsyn in lipid dynamics during enucleation is independent of hypothesized SNARE functions.^103^ However, if αsyn is involved in this cellular phenomenon, it is non-essential or interchangeable with beta or gamma synucleins, as only minor phenotypic abnormalities of erythrocytes are observed in α-syn-knockout models.^99^

## Soluble oligomers devoid of lipid

**Fig. 1:**
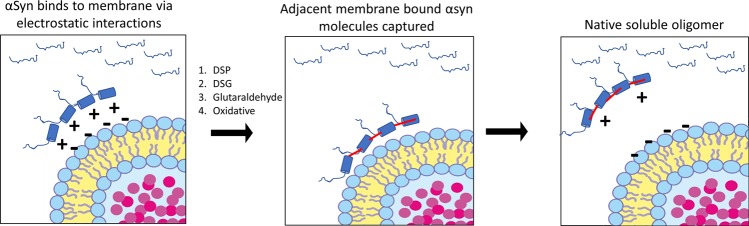
Endogenous soluble oligomers are inextricably associated with lipid/vesicle binding. Depicted is an possible explanation for the detection of a soluble αsyn oligomer. In the cytoplasm, αsyn exists in an equilibrium between a disordered slightly compact monomer and membrane-bound α-helix confirmation. The N-terminus of αsyn binds to vesicle membranes via electrostatic interactions and adopts an α-helix structure. αSyn most likely binds to localized areas of vesicle surfaces with lipid-packing defects. Normally, in the cell ~5–10% of αsyn is interacting with vesicle surfaces. The same percentage is also proposed for soluble oligomers. Covalent bonds between adjacent αsyn molecules capture the confirmations bound to the vesicle surface. Covalent modification of amino-acid residue side chains, especially lysine, following chemical crosslinking neutralizes a portion of αsyn charge required for membrane binding. The captured species could then retain the membrane-bound confirmation and enter the aqueous phase for subsequent detection. Thus, endogenous soluble functional oligomers are unlikely, in agreement with several studies. Instead, endogenous oligomers may represent confirmations of membrane-bound αsyn. This hypothesis makes lipid-syn interactions at the membrane a crucial mediator of pathology initiation. DSP dithiobis(succinimidyl propionate), DSG disuccinimidyl glutarate

There is good evidence of a naturally occurring metastable soluble αsyn oligomer (i.e., tetramer) that is devoid of vesicle/lipid binding.^8^ However, the existence of a soluble αsyn tetramer is based mostly on results from crosslinking experiments.^8,48,104–106^ The interpretation of crosslinking experiments is non-trivial. αSyn tetramers are captured when using a permissive chemical cross-linker with spacer arm length (DSG spacer arm length 7.7 Å) and perhaps not with a shorter spacer arm (formalin spacer arm length ~2 Å).^107^ A milieu of progressively larger oligomers are formed and captured even when purified recombinant αsyn is incubated with glutaraldehyde.^108^ The successful detection of an αsyn tetramer in tissues and cells is dependent on sample preparation conditions and can be detected when cells remain intact prior to crosslinking or when tissue lysates are kept highly concentrated.^107^ Indeed, purification of αsyn prevents the detection of a soluble tetramer further suggesting a cofactor is required and this factor is likely of lipid origin.^109^ Although the question remains which lipid cofactor might be responsible for the tetramer formation, the tetramer and αsyn–lipid interactions are inextricably linked. This is highlighted when recently a transgenic mouse model (called “3K”) of tetramer deficiency was generated by introducing 3E– > K mutations in αsyn’s lipid-binding N-terminus.^48^ These 3K mice exhibit aggressive αsyn aggregation, loss of an apparent tetramer, and a motor phenotype that has some semblance to Parkinson’s disease. The lipid-binding domain was mutated in the 3K mice and correspondingly lipid interactions were enhanced^48^ and similar to what was observed with similar mutations in cells.^110^ In both mice and cells, mutated 3K αsyn clustered around vesicles and intact tissue crosslinking captured less soluble tetramer.^48,106,110^ Was this due to less tetramer or alternatively less soluble tetramer? The results could be explained either way, but if the captured αsyn species is truly an insoluble tetramer, one would expect less detection in the soluble fraction, as the authors observed. Indeed, the results appear to fit a scenario where folding on the vesicular membrane is driving αsyn pathology without the need for a soluble tetramer. (Fig. 1)

Detection of endogenous oligomers, including the tetramer, could be explained by compartmentalized αsyn residing on the vesical membrane (Fig. 1). Membrane interactions occur through electrostatic forces between positively charged lysine residues of αsyn and negatively charge phosphate group of lipids.^41,75,81,111^ Interestingly, the same crosslinking chemicals used to capture tetramers chemically modify lysine side chains of αsyn and neutralize their charge.^112^ Chemically modifying the lysine side chains during tissue crosslinking would presumably disrupt membrane binding, because it neutralizes the required electrostatic charge of lysine residues. Therefore, captured oligomers would dissociate from the membrane and would be detected in the soluble fraction, producing the characteristic gel-shift of the αsyn tetramer.^106^ To highlight this concept, the neutralization of αsyn charge by chemical cross-linkers is routinely used to enhance the retention of αsyn on polyvinylidene difluoride membranes during western blotting protocols.^112^ Together, adjacent αsyn molecules bound to intracellular vesicle surfaces (i.e., compartmentalized) might disassociate into the soluble fraction once chemically modified by the crosslinking reagent. This phenomenon would also help explain why the soluble tetramer has similar intermolecular n-terminal structure as the membrane-bound form.

The apparent soluble tetramer may be stabilized by covalent bonds formed in the oxidative environment of the erythrocyte (i.e., the source from which it was originally isolated). Biochemical characterization of a putative αsyn tetramer was mostly done using erythrocyte derived αsyn.^8^ Erythrocytes have millimolar concentrations of hemoglobin. Hemoglobin oxidatively catalyzes the formation of intramolecular dityrosine bonds resulting in a mixture of αsyn dimers and tetramers.^113^ Dityrosine crosslink formation occurs rapidly^113,114^ and would likely occur to some extent during αsyn purification from erythrocytes. αSyn in erythrocytes associates with vesicles^101,102^ and stable dityrosine αsyn occurs in clinical blood samples.^115^ Together, it is probable that stable αsyn oligomers isolated from erythrocytes are due to oxidative crosslinking of adjacent αsyn molecules bound to vesicle membranes. Heat denaturation irreversibly abolished the tetramers α-helix structure, indicating that the captured configuration was not in equilibrium but instead was a stabilized structure originating from the tissue (i.e., vesicle bound). Lipid binding of the tetramer was enhanced when compared with the monomer, further suggesting it retained a lipid-binding confirmation.^8^ Removal of lipids with Lipodex 1000 did not affect the tetramer detection, suggesting the tetramer was not associated with any stabilizing lipid structure. Stabilization of small oligomers via oxidative crosslinking can prevent progressive aggregation and might explain why the stable tetramer appears to resist aggregation.^8,116^ Together, it is likely to be that the stable soluble αsyn tetramer purified from erythrocyte is a covalently stabilized membrane-bound confirmation similar to that captured using exogenous crosslinking agents.

Soluble αsyn oligomers have been detected using several imaging techniques. Förster resonance energy transfer (FRET) is a powerful technique used to determine intermolecular distances between molecules with 1–2 nm spatial resolution.^117^ FRET has been employed to study various aspects of αsyn oligomerization mostly in vitro^9,77,118–120^ but also ex vivo^121^ and in vivo.^122^ In vitro, purified αsyn forms distinct oligomer conformers, which then can spontaneously convert to protease resistant and toxic αsynO-β.^12^ Biomolecular fluorescence complementation (BiFC) technique uses fluorescent constructs to determine protein–protein interaction. BiFC constructs have been used to study αsyn aggregation in vivo;^123,124^ however, the resolution of this technique cannot differentiate between small oligomers and complex formation (i.e., membrane bound). The method detects diffuse staining in neurons lacking pathology, suggesting either small aggregates or close association of αsyn molecules normally within the cytosol.^124^ αSyn comes into close proximity around synaptic vesicles and possibly forms multimers on the membrane.^15^ Other BiFC techniques employing photoactivatable fluorescent molecules can increase the spatial resolution to several nanometers,^125^ but this type of imaging has yet to be done with αsyn.

## Soluble disordered monomer devoid of lipid

There is also good evidence that αsyn exists predominantly as an intrinsically disordered monomer in the cytosol.^10,14,126–130^ αSyn purified from *Escherichia coli* behaves as an intrinsically disordered protein with a large stokes radius,^129^ which may be why monomeric αsyn appears to have greater mass in some assays.^126^ Non-denaturing purification procedures from several tissue sources also produce a disordered monomeric αsyn.^126^ A disordered soluble monomer has been observed directly using in-cell nuclear magnetic resonance (NMR) imaging techniques.^14^ Specifically, investigators transfected cells with recombinant αsyn labeled with ^15^N isotope to monitor individual αsyn molecules within the living cells. Results showed that the majority of monomeric αsyn maintained a disordered confirmation in the cell, while becoming slightly more compact than in free solution, probably due to molecular crowding.^14^ The compact structure observed in vivo likely prevents spontaneous aggregation in the cytosol.^131^ Although this is compelling evidence that the majority of αsyn in the cell occurs as a disordered monomer, the result does not rule out the existence of a tetramer. A tetramer that existed at low concentration would not be detected and it is possible that the recombinant αsyn behaved dissimilarly to endogenous αsyn. Importantly, this study demonstrated that the majority of αsyn in the cell is cytosolic and monomeric, and suggests that membrane interactions are likely transient and highly dependent on local environment (e.g., nerve terminal). Correspondingly, it would be interesting if αsyn persists as a monomer at axon terminals where it’s vesicle interactions are more prominent than in the cell body.^73,132^ The α-helix conformation αsyn was recently described in HELA cells using FRET, where it was demonstrated that αsyn assumes several confirmations when interacting with vesicle surfaces.^133^ Considered together, the majority of αsyn in the cell exists as a relatively compact disordered monomer and adopts an α-helix structure when interacting with vesicle membranes. The native state of αsyn may not include an oligomer, whether free and soluble, or vesicle bound.

## Aberrant vesicle binding progresses to pathology

**Fig. 2:**
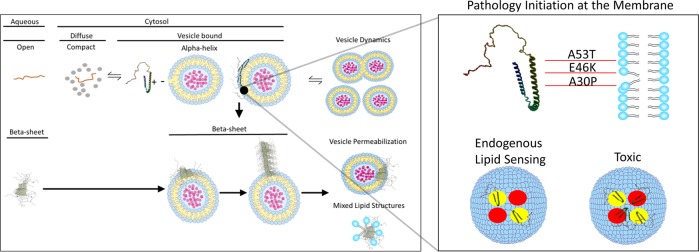
Potential role of lipids in αsyn aggregate pathoetiology. In the cell, αsyn is partitioned between aqueous phase and the lipid phase via transient interactions at the vesicle surface. Endogenous αsyn probably exists in several states, including a compact monomer and a vesicle-bound monomer with an N-terminal α-helix structure. Folding αsyn monomers on the vesicle surfaces likely plays a non-essential or redundant role in vesicle dynamics. β-Sheet confirmation of αsyn may begin at vesicle surfaces. Toxic effects of β-sheet oligomers included vesicle permeabilization or the formation of toxic mixed lipid–protein structures. Pathology initiation might involve specific configurations of αsyn folding onto a variety of membranes. Altered lipid-sensing properties by known disease-causing mutations (e.g., A30P, E46K, and A53T) might alter the affinity of αsyn for certain vesicle lipid components (depicted as yellow and red circles), or change the spatial arrangement of αsyn molecules on the vesicle surface. Resulting β-sheet oligomers may have different toxic or prion-like properties based the physiochemical details of the initial pathology development

Assuming monomeric αsyn is interacting with vesicle membranes, and remains monomeric at the vesicle surface under normal circumstances, how might pathology begin? (Fig. 2). One possible scenario involves vesicle surfaces acting as two-dimensional (2D) reactors that promote pathogenic intermolecular interactions of αsyn.^91,134–136^ In the cystosol, αsyn remains monomeric and in a slightly compact configuration. Transient interactions with vesicle surfaces induce a conformational shift, but not necessarily oligomerization, and concentrate αsyn molecules on the vesicle surface. This focal point on the vesicle surface is where opposing αsyn molecules bind and might serve as the molecular origins for Lewy pathology. Numerous cellular and genetic factors converge at this focal point in such a way that creates an environment conducive for the initiation of pathogenic αsyn aggregation. Studies using sonicated αsyn PFFs suggest that once the αsynO-β is present, progressive aggregation and toxicity follow.^7,137^ Yet, studies that utilize PFF’s to assess pathology are bypassing pathology generation and may be recapitulating downstream pathological events. Therefore, the use of PFF’s to study synucleinopathy is likely to give valuable insight into the progression of these diseases and perhaps are not suitable to study the initiation of the disease.

At the membrane, αsynO-β might act similar to a “molecular shovel” inserting itself into the membrane with destructive, toxic, consequences.^2,138^ Together with lipid/vesicle interactions at the center of αsyn function, a route to pathophysiology might be the collapse of clustered lipids/vesicles into a pathological inclusion. Similar to a massive star transitioning to a black hole, at some point pathological αsyn and lipids form a critically dense structure, and compact to form a Lewy body. Interestingly, the architecture of the Lewy pathology supports this interpretation.^139–144^ Neuropathological examination of patient brains shows a mixture of αsyn staining in neurons that consist of a pale diffuse, punctate irregular shape (i.e., uneven distribution), discrete body (i.e., pale body), and a massive dense structure with a pale core (i.e., Lewy body).^139^ These structures have been hypothesized to be snapshots of a pathological process with Lewy bodies being the result. Indeed, pale bodies contain a mixture of granular and vesicular structures and are often found near mature LB.^141,145^ Using a lipid centric view, punctate irregular “early” pathology might represent the initial vesicle clustering^48^ or lipid droplet formation^47^ before the characteristic pathology develops. Overexpression of αsyn in yeast models produces lipid only inclusions, lacking the filamentous αsyn that is the hallmark of synucleinopathies.^146^ Lipids have been identified as a core component of Lewy pathology^33,147,148^ but this has largely been ignored and the pathology is often considered “proteinaceous.”

Recent work by Shahmoradian and colleagues^149^ provided substantial evidence that Lewy pathology consists of compacted lipid components from a variety of organelles with αsyn oligomers interspersed. Their work strongly suggests that Lewy pathology is actually an inclusion of fragmented lipids, for which αsyn–lipid interactions play a causative role. Ultrastructural characterization of Lewy pathology showed tubule vesicular, fragmented membranous, and mixed lipid–protein structures, all of which can be formed from αsyn interactions with vesicle membranes. Electron dense structures, consistent with lysosomes, were also observed throughout Lewy pathology.^149^ Lysosomes are central mediators of lipid metabolism^150^ and the conspicuous presence of lysosomes surrounded by fragmented membrane structures strongly suggests a deficit in lysosomal/autophagic pathways, specifically the removal of lipid membranes. Large-scale genome-wide association studies have implicated lysosomal/autophagy pathway in several neurodegerative diseases, including synucleinopathies.^151,152^

A lipid-centric view of Lewy pathology is transformative in that it helps unify and identify disease-causing pathology of several molecular origins. Several neurodegenerative diseases are currently characterized by protein aggregation, when instead we may be missing the lipid components that are the core of the pathology. For example, clinical cases resembling synucleinopathies are documented without the presence of Lewy pathology (e.g., Parkin mutations with early-onset Parkinson’s disease), as measured by αsyn staining.^153^ The presence of lipid inclusions in the absence of αsyn are not generally considered when examining patient tissues.

## Determining αsyn–lipids interactions in living cells

αSyn interactions with lipids and vesicles has been investigated mostly in vitro and needs to be characterized in living cells. There are several promising strategies to start understanding lipid–αsyn interactions. The first strategy uses synthetic bifunctional lipids to directly determine lipid–protein interactions.^154,155^ This strategy offers flexibility with analysis and offers unambiguous evidence of direct αsyn–lipid interactions in vivo. Captured lipid–αsyn molecules can be subsequently labeled or purified for downstream analysis. Labeling the structures will help determine where αsyn–lipid interactions are most relevant in the cell. Purification of the structures with subsequent analysis by liquid chromatography–mass spectrometry could determine specific αsyn proteoforms involved with pathological lipid interactions, as well as global analysis of other proteins that are involved. However, the drawback to this strategy is the investigator can only assess one specific lipid species at a time and a synthetic bifunctional lipid must be available or developed for application. Recently, a bifunctional analog of glucosylceramide, a lipid implicated in synucleinopathy,^156^ has become commercially available and could aid in these studies.

A shotgun lipidomic analysis may also be useful, but because of the complexity of whole-cell lipid determination, the data may not give insight into the localized αsyn–lipid interactions that precede pathology formation. Recently, a shotgun lipodomic analysis was conducted on various αsyn mutant models and αsyn was found to have an effect on lipid metabolism.^49^ However, it is difficult to draw distinct conclusions or find drug targets based on the description of a total lipid species. The resulting information is most useful in implicating lipid metabolism or catabolism pathways, and not the characterization of the specific localized lipids that may be involved with initiating pathology. To find a disease-relevant target, a focused lipodomic approach looking at specific organelles, or better yet, early Lewy pathology, will be the most illuminating. Lipodomic arrays can also be used to screen many lipid–protein interactions; however, they have the disadvantage of not representing in vivo binding conditions.

A key question remains: at what point during its interaction with cytoplasmic membranes and extracellular vesicles does αsyn adopt a pathological confirmation? To answer this question, one needs to consider the membrane as a chemical reactor favoring molecular encounters.^134^ This is the consequence that restrains monomeric or low-molecular-weight oligomeric αsyn diffusion from a three-dimensional to a 2D space upon interaction with the plasma membrane or extracellular vesicles plane.

## Conclusions

Evidence for a soluble αsyn oligomer might be best explained by folding intermediates on the plasma or vesicle membranes that remain soluble for subsequent extraction and detection. This interpretation does not require a soluble functional oligomer and seems to fit much of the experimental data. The distinction between a soluble native oligomer and vesicle-bound oligomers/folding intermediates is important, because it clarifies the origins of pathological aggregation of αsyn. With this perspective, determining the molecular details of αsyn–vesicle/lipid interactions is important for understanding the endogenous origins of synucleinopathy. Although there is consensus that aggregation of αsyn is associated with neurological disease, the precise molecular origin of the aggregate pathology remains a mystery.
